# Accuracy Evaluation of the Unified *P*-Value from Combining Correlated *P*-Values

**DOI:** 10.1371/journal.pone.0091225

**Published:** 2014-03-24

**Authors:** Gelio Alves, Yi-Kuo Yu

**Affiliations:** National Center for Biotechnology Information, National Library of Medicine, National Institutes of Health, Bethesda, Maryland, United States of America; Queen's University Belfast, United Kingdom

## Abstract

Meta-analysis methods that combine 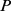-values into a single unified 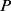-value are frequently employed to improve confidence in hypothesis testing. An assumption made by most meta-analysis methods is that the 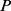-values to be combined are independent, which may not always be true. To investigate the accuracy of the unified 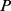-value from combining correlated 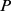-values, we have evaluated a family of statistical methods that combine: independent, weighted independent, correlated, and weighted correlated 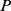-values. Statistical accuracy evaluation by combining simulated correlated 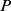-values showed that correlation among 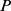-values can have a significant effect on the accuracy of the combined 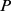-value obtained. Among the statistical methods evaluated those that weight 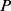-values compute more accurate combined 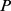-values than those that do not. Also, statistical methods that utilize the correlation information have the best performance, producing significantly more accurate combined 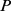-values. In our study we have demonstrated that statistical methods that combine 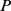-values based on the assumption of independence can produce inaccurate 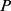-values when combining correlated 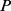-values, even when the 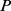-values are only weakly correlated. Therefore, to prevent from drawing false conclusions during hypothesis testing, our study advises caution be used when interpreting the 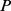-value obtained from combining 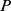-values of unknown correlation. However, when the correlation information is available, the weighting-capable statistical method, first introduced by Brown and recently modified by Hou, seems to perform the best amongst the methods investigated.

## Introduction

Meta-analysis methods that combine 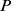-values into a single unified 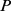-value are commonly used to rank or score a list of hypotheses [1]. For each hypothesis tested, the 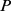-values to be combined are often acquired from studying different features associated with the hypothesis or from using different data analysis methods (DAM) to analyze a chosen feature. Either approaches conducted to test the same list of hypotheses assign an overall 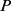-value to each hypothesis tested. These 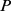-values are then usually sorted, with the most significant result ranking first in the list. Given that different features may not be completely independent and that different DAMs may share protocols and use similar information, it is likely that the 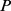-values obtained for a hypothesis are correlated.

Most 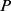-value combining methods assume that the 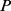-values to be combined are independent or weakly correlated [2], [3]. When the unified 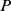-value is computed by combining correlated 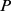-values, without properly taking into account the correlation, there can be notable effects in the significance assignment of the hypothesis tested. As the 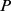-values to be combined are possibly correlated, it is important to investigate the effect that correlation has on the unified 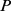-value. The current study is designed to evaluate the accuracy of the unified 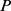-value computed by combining (positively) correlated 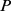-values using some commonly applied statistical methods. By 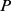-value accuracy, we mean how well on average does reported 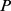-value agree with the one-sided cumulative distribution function of the random variable (associated with the null hypotheses tested) at the critical region. In other words, accurate 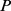-value means that when one controls type-I error rate at a level 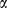, the type-I error rate is really controlled at the level 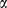. To keep this paper focused, we will not provide a lengthy introduction. For methods that we will evaluate, more details are provided in the Methods sections. For others, we will only provide the readers with appropriate references.

Several studies have been performed to evaluate methods that combine independent 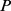-values [4]–[10]. For example, Rosenthal has evaluated nine methods for combining 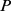-values and has summarized advantages, limitations and applications for each method [4]. Loughin [5] has also conducted a systematic comparison of methods for combining 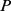-values and recommended practitioners to choose a method based on the structure and expectation for the problem being studied. Recently, Whitlock [6] has showed that the weighted Z-method has more power and precision than Fisher's test. In other studies, Chen [8] as well as Chen and Nadarajah [9], have shown that either the generalized Fisher method due to Lancaster or a special case of Lancaster's test outperform the weighted Z-method, while Zaykin [10] has shown that the weighted Z-method has similar power to Lancaster's method when the weights are selected to be the square roots of sample sizes.

As for combining correlated 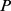-values, only few studies have been conducted to evaluate the accuracy of the unified 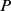-value computed by existing statistical methods [11], [12]. Evidently, more comprehensive investigations that incorporate different methods, encompass a wide range of correlation strength, and have a large number of simulations can further our understanding on the effect of correlation has on computing a unified 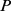-value. To advance towards this direction, we systematically investigate a family of statistical methods for combining 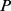-values. Because we are interested in combining 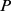-values obtained from the right-tailed tests, we have limited our study to methods that combine 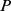-values based on the normal distribution (e.g. Stouffer's method) and on the Chi-square distribution (e.g. Fisher's method), the general purpose method and the right-tail method recommended by Loughin [5]. The two aforementioned methods, aside from being frequently used to combine 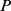-values, are useful and important to study for the following reason. Both methods mentioned have variations that weight 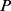-values while computing the combined 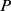-value: Lipták, Good and Bhoj methods [13]–[15], and variations that take into account the correlation among 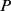-values: Hartung and Hou methods [16], [17]. In addition, all methods mentioned above either have closed-form formulas, i.e., distribution functions, or approximation formulas that can provide the unified 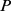-value with minimum computation cost.

In summary, our study presents an accuracy evaluation of the unified 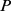-value obtained from statistical methods designed to combine independent, weighted independent, correlated, and weighted correlated 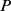-values. We have evaluated the accuracy of the unified 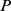-value from combining positively correlated 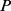-value vectors with correlation among 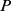-value vectors in the range 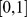. Our results show that methods designed to combine independent 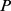-values but with the capability of assigning weights to 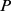-values perform better than methods that combine independent 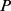-values without weights. Also methods that take into account the correlation between 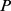-values perform significantly better than methods designed to combine independent 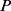-values. Based on this study, the method first introduced by Brown [18] to combine correlated 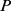-values and later adapted to include weights by Hou [17] is the best performing one amongst the methods investigated.

## Methods

The main task of combining 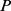-values is described below. Given a list of hypotheses 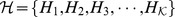, let each hypothesis have 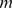 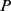-values associated with it. These 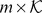 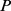-values can be organized as 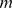 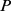-value vectors, 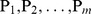, each having 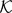 components. Each 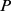-value vector may result from analyzing one out of 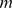 different features of every hypothesis or may be from analyzing a single feature using one of the 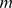 different DAMs. The 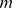 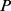-values associated with hypothesis 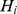 are 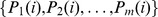. Given those values, one needs to combine them to form a single unified 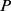-value. This scenario can occur in many applications. As an example, when different studies are performed to test a set of genetic loci for allelic imbalance [19], the number of genetic regions tested will correspond to the number of hypotheses 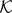 and each region will carry with them 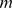 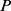-values, one from each of the 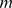 studies. To fairly rank these possible 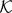 regions, for each region one would need a unified 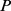-value resulting from combining the 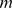 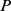-values associated with it. For database search based peptide identification using mass spectrometry, it is possible to analyze the data using multiple analysis methods. Here for each experimental spectrum, the number of hypotheses tested 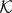 equals the number of scored peptides in the database and each peptide receives a 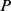-value from each of the 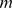 analysis methods. To fairly rank the candidate peptides, it is again natural to combine the 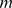 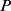-values associated with each scored peptide [3] to reach a unified 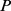-value. In the sequence homology detection where multiple motifs are used as a query to a sequence database, it is often needed to combine the 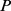-values, each from one of the 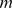 motifs, to assign the statistical significance to a sequence in the sequence database [2]. In this case, 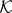 is the number of sequences in the database, while 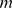 is the number of motifs used as the query.

To make the notation uniform, we will use 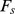 and 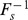 to represent the cumulative distribution and inverse cumulative distribution. When the subscript 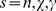, 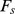 represents respectively the cumulative Normal, Chi-squared, and Gamma distributions. All the parameters of these distributions will be shown as arguments enclosed by a pair of parentheses following the symbol 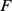.

### Combining Independent *P*-values

We begin this subsection with a brief introduction of Stouffer's (Z-transform test) and Fisher's (Chi-square test) methods. Generalizations of both methods to combine weighted 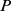-values are also described.

#### Method 1

The combined Z-transform test was first used by Stouffer *et al*. [20] and later generalized to include weights by Lipták [13]. Under the null hypothesis, the 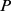-values are uniformly distributed between [0,1]. Given a list of 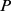-values 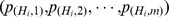 associated with a given 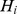, one transforms the 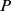-values to a new variable 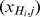 by a simple transformation
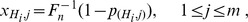
where 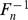 stands for the inverse of the cumulative normal distribution. For the Z-transform test the distribution function used is the standard Normal (Gaussian) distribution with probability density function given by
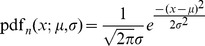
with parameters 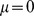 and 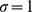.

Stouffer's way to combine the above 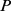-values is by defining a new variable
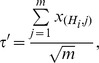
which is also Gaussian distributed with 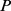-value given by the formula
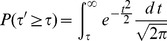
(1)


A generalization of the above equation that assigns weights (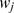) to the variable 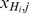 is know as the weighted Z-transform test [13]

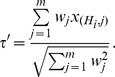



The variable of the weighted Z-transform 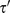 also follows Normal distribution, and the formula for the 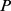-value is also given by eq. (1).

#### Method 2

Fisher's method [21] is one of the most used method to combine independent 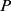-values. The combined Fisher 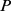-value is obtained through the following variable:
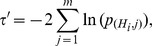
which follows a Chi-squared distribution 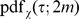 with 2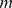 degrees of freedom. Computing the unified 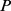-value using the Chi-squared distribution is not the most efficient approach because of the significant computational cost in calculating the cumulative distribution 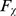. A more efficient way to obtain the unified 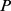-value has been proposed [2], [3], where the unified 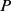-value of 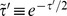 has a closed form given by
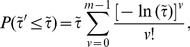
(2)or in terms of the 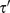 variable
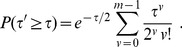
(3)


Note that as 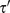 increases 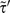 decreases and vice versa.

Fisher's method does not assign weights to the 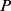-values to be combined. However, when information is available regarding how 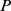-values were obtained, it might be beneficial to weight 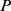-values. Lancaster *et al*. [22] addresses this issue by replacing the random variable 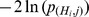 with 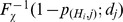, a variable following a Chi-squared distribution with 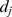 degrees of freedom not necessarily equal to two.

In Lancaster's procedure, summarized below, one can exploit the equivalence between the Chi-squared distribution 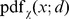 and the gamma distribution 
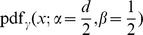
 to reach a different weighting generalization. For hypothesis 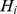, the variable 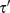 can now be written as
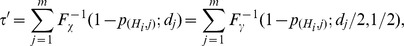
which evidently follows a Chi-squared distribution with 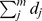 degrees of freedom. In the expression above, Fisher's method is recovered by setting 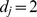 for all 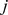. Another way to incorporate weights is to keep 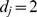 while retaining a general 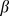 value. Specifically, one may choose, with 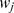 being the weight factor, to use the following new variable
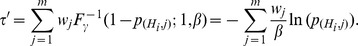



The 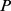-value for 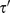 can be easily evaluated using the same technique as that in [3] and is given below
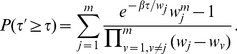
(4)


Interestingly, with 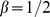, eq. (4) corresponds to the unified 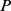-value of multiplying weighted independent 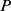-values obtained earlier by Good [14]. This can be seen by the following observation. Good defined his variable
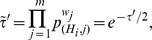
and the corresponding 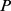-value is given by



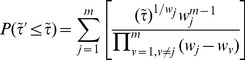
(5)


When expressed in the variable 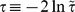, we easily see that



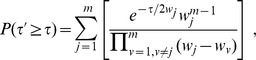
in agreement with eq. (4) when 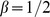.

A question that arises naturally when using methods such as the weighted Z-transform's test, Good's test, and Lancaster's test is how to obtain the optimal weights (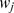)? This difficult question has been raised and it was suggested that the choice of weights may vary by cases [23]. Existing methods to assign/estimate the weights include, but are not limited to: (1) weight in proportion to the reciprocal of the variance estimated from each study [6], (2) estimate the weights from one's prior belief about a method or feature [24], (3) select weights to stabilize the variance of the combined test statistics [25], and (4) use weights that improve the testing power [26]. Because there is no universal procedure to compute the optimal weights to be used, in this study the weights, when used, were randomly generated and normalized to sum to one (see Table 1).

**Table 1:** Breakdown of Methods Used to Combine 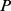-values Investigated.

Method Name	Ref. number	Eq. number	Acc. weights	Nor. weights	Account for corr.
Fisher	[21]	3	no	none	no
Stouffer	[20]	1	no	none	no
Bhoj	[15]	6	yes	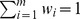	no
Good	[14]	5	yes	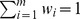	no
Lipták	[13]	1	yes	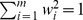	no
Hartung	[16]	9	yes	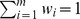	yes
Hou	[17]	14	yes	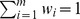	yes

The first column of the table provides the names of the methods used to combine 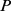-values investigated in our study. The second column lists the reference number cited in this paper for the publication (Ref) corresponding to the method used. The third column provides the equation number for the method distribution function used to compute the formula 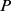-value. The fourth column indicates if a method equation can accommodate (acc.) weight when combining 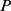-value. The fifth column gives the normalization (nor.) procedure used to normalize the weights. Finally, the last column conveys the information about a method's capability to account for correlation (corr.) between 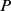-values.

There are also two apparent problems with Lancaster's eq. (4) and Good's eq. (5). The first problem is that the weights used can't be identical, otherwise singularities can occur [14], [15]. Second, if the difference between some of the weights are small, numerical instability can occur [15], [17], [27]. In order to address the problem of numerical instability associated with identical and almost identical weights, Bhoj [15] suggested an approximation using a linear combination of 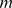 gamma density functions (with 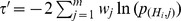)
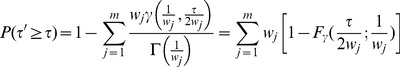
(6)where 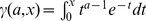 is the incomplete gamma function and 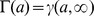 is the gamma function. Although the approximation provided by Bhoj does reduce to Fisher's distribution when the weights are all equal and does not encounter singularities when weights are identical or nearly identical, this approximation does not lead to Good's distribution when the weights are all different. A recent publication [27] has provided an analytical formula that not only is numerically stable when combining 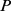-values with nearly degenerate or identical weights but also correctly reproduces Fisher's and Good's results as limiting cases.

### Combining Dependent *P*-values

In this subsection we summarize two statistical methods that are generalizations of Stouffer's test (Z-transform test) and Fisher's test (Chi-square test) that attempt to account for the correlation among 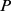-values to be combined.

#### Method 3

Hartung [16] incorporates the correlation among 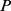-values via introducing in the Z-transform test (eq. (1)) the correlation-matrix, with elements 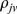 computed from the variable pairs 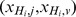, and by defining a new variable
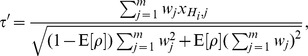
where
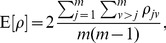
(7)and
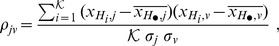
(8)where 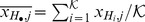 is the average value of 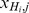, 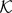 is the total number of hypotheses tested, and 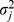 is the variance of 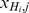.

The 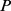-value for 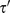 is then approximated by the standard Normal distribution
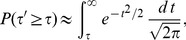
(9)which nevertheless becomes exact in the two extreme limits of 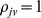 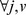 and 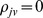 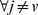. Although in general the distribution of 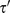 is only approximately normal, it is arguable that ignoring correlation can cause more damage to the combined 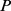-value than the deviations from normality. Applications and extensions of Hartung's idea can also be found in more recent publications [12], [28].

#### Method 4

Following Satterthwaite's procedure [29], there have been some attempts, when combining correlated 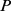-values, to obtain approximate unified 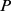-value for the Fisher's variable (no weight) [18], [30] and for the Good's variable (unequal weights) [17]. The main idea of Satterthwaite's procedure is to equate the first two moments of the uncharacterized distribution to that of a Chi-squared distribution. Brown [18] and Kost *et al*. [30] tried to approximate the distribution of the Fisher's variable
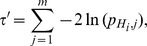
and Hou [17] the distribution of Good's variable
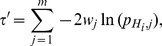
to that of a Chi-squared distribution 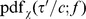, with 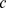 being a scale factor to be determined.

The expectation value (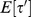) the variance (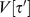) of 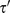 by formal operation are given respectively by
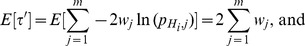
(10)

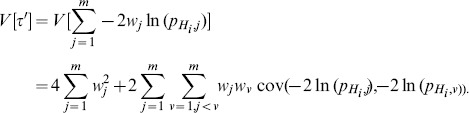
(11)


On the other hand, the expectation value and variance of 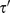 using 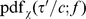 yields
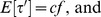
(12)

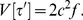
(13)


Equating (10) to (12) and (11) to (13) yields
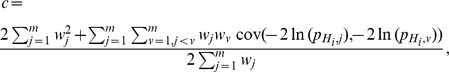
and
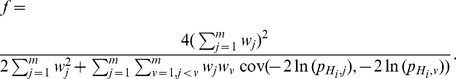



The covariance (cov) term used above was first estimated by Brown [18] and recently an improved estimation (through numerically tabulating the covariance as a function of the correlation and then performing polynomial fits) was provided by Kost and McDermott [30]

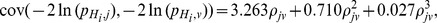
where 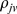 above is the correlation between 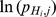 and 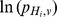. The 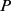-value for 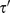 is then approximated by that of a Chi-squared distribution
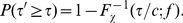
(14)


Equation (14) reduces to Fisher's formula eq. (3) when the 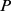-values are independent and the weights are all same. However, the above equation does not reduces to Good's formula eq. (5) when the 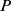-values are independent and each carries a different weight.

### Generating Correlated P-value Vectors

By definition, the 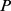-values of null hypotheses should be uniformly distributed between 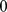 and 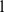, which is often assumed by methods of combining 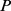-values. However, the uniformity of 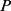-values, when assigned by available statistical tools to a group of null hypotheses, is often lost. This would handicap the efficacy of methods for combining 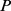-values from the start. To eliminate the effect of nonuniform null 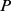-values from our evaluation, we enforce the quasi-uniformity of null 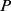-values by first constructing a starter 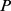-value vector 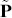 of size 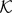 with the 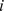
th element 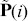  =  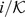, for 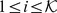. (See next paragraph for more details.) This guarantees an even sample of the 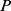-values (in the range from 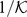 to 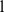). To achieve correlations of various strengths, we have used 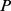-value vectors, each of which is obtained via permuting (pairwise) the elements of a fixed vector, the starter vector with a small perturbation, by a randomly chosen number. The basic idea is that when the number of pairwise permutations is not large, the resulting 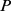-value vectors will be correlated to the fixed vector and will be correlated among one another. It is worth pointing out that this approach does not generate correlations with a *prescribed* strength: even with the same number of random pairwise permutations of the vector elements, the correlation between any pair of such *permuted* vectors does not have a fixed strength. We believe this is closer to the real-world scenario than having a fixed correlation strength among the 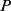-value vectors. The value of 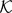 should not matter in terms of testing whether a method can provide accurate combined 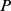-value. If a small 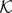 is used, however, the combined 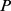-value will have a large statistical fluctuation that may reduce the resolution of the comparison. On the other hand, making 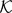 large causes a long computational time. We find that using 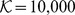 yields enough separations among methods tested without significantly slowing down the computation.

**Figure 1:**
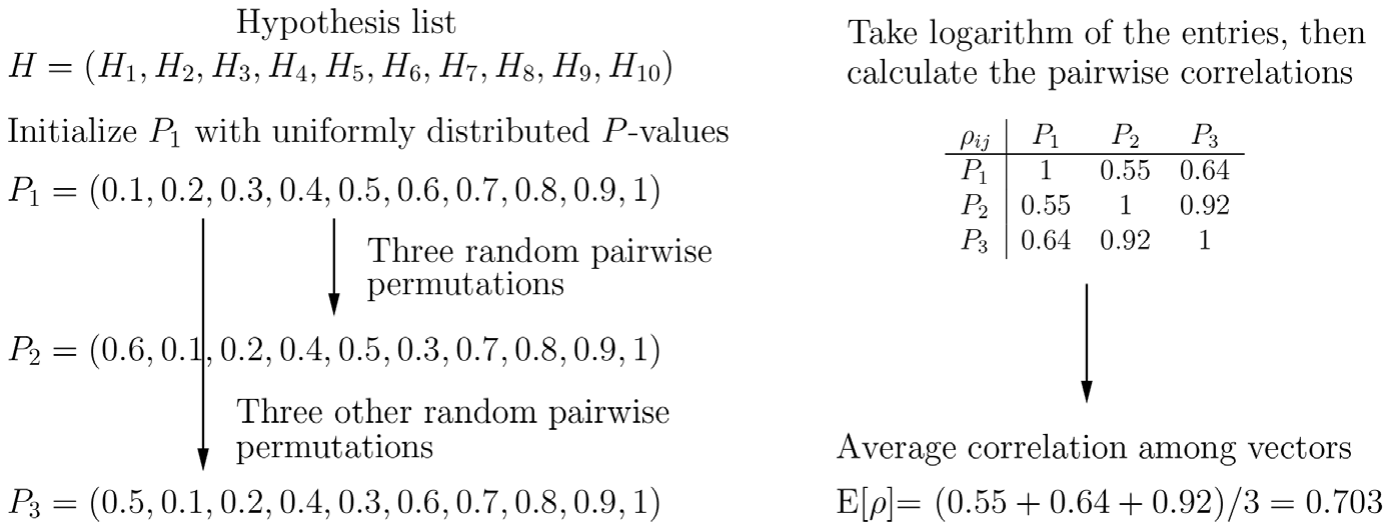
Example workflow of generating correlated 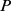-values and pairwise correlations. In this example figure, 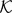 is 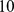, the number of 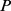-value vectors is 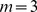, the number of pairwise permutations 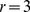, and the perturbations 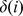
s are set to zero for clarity and simplicity. The resulting pairwise correlations by using 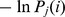 in place of 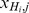 are displayed in a symmetric matrix form.

For each method investigated, we have performed a simulation of 500,000 realizations, each of which was conducted as follows. First, pick a random positive integer 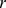 with 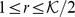. Second, generate the first 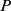-value vector 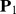 by adding a small random perturbation (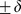) between 0 and 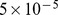 to each vector element of 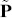: 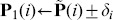. Evidently, by increasing the upper bound for 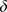, one will produce 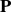-values with larger variations from exactly uniform distribution. In the third step, generate more size-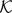 vectors 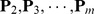 and initialize them to 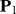. For each vector generated, its vector elements are pairwise permuted 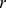 (chosen at the first step) times. After that using 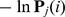 in place of 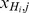 the pairwise correlation 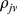 was computed using eq. (8) and the average correlation E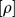 among vectors was computed using eq. (7). This work flow is illustrated in Figure 1 with 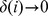 for simplicity. The constructed random 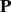-value vectors 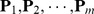 were then combined to obtain a unified 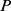-value vector (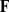) using the various methods listed in Table 0. Once the unified 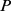-value vector (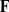) was calculated, its elements were sorted in increasing order and it was then compared against the rank (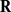) vector, whose element is obtained by dividing the rank of a 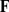 element by 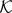, i.e., 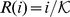 for 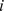 ranging from 1 to 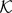. We shall call 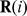, the 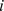
th element of the rank vector, the *normalized rank* of rank 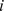.

### Statistical Accuracy Evaluation of the Combined *P*-value (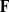)

**Figure 2:**
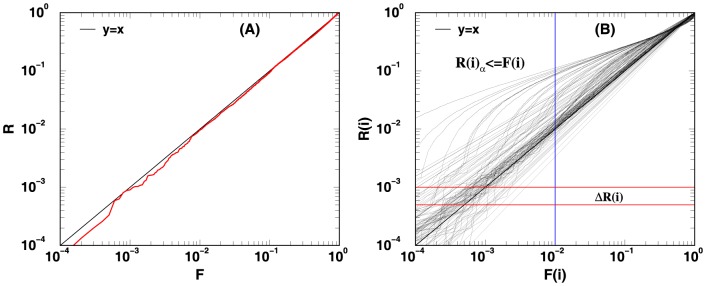
Log-log plot of the unified 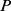-value vector 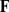 versus the rank vector 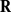. The curves in panels (A) and (B) were obtained from combining the 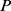-values of four 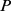-value vectors, each of size 10,000, using Stouffer's method. In panel (A), the red circles show the scatter plot of normalized rank versus computed 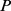-value from a randomly picked iteration (realization) of very weak average correlation. It is through curves like the one displayed in panel (A) that enables one to calculate the average sum of squares error using eq. (15) and the distance measure using eq. (16). Panel (B) shows 1000 curves, each of which is obtained from performing the same task as that leads to the curve in (A) but with different average correlation strengths. The lines that go significantly above 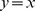 line are from cases with stronger average correlations. They yield unified 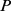-values that are much exaggerated perhaps due to the fact that the Stouffer's method does not account for correlations. By averaging the normalized rank 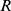 along the blue line (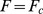) yields the value 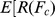 (see eq. (17)). By shifting the blue line to different 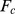 values renders the entire 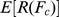 versus 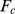 curve. The red horizontal line illustrates the case when 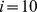 (or normalized rank 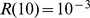). By averaging the 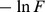 values along this line, the 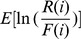 value is obtained for 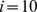 by simply adding 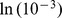 to the averaged value (see eq. (18)).

If a method yields a unified 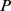-value vector 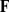 agreeing with 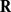, the scatter plot of 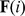 versus 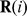 should produce a straight line with slope one and intercept zero [31]. It is also important to mention that the smallest computed 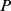-value is expected to be inversely proportional to the sample size, which for the current case is of the order of 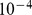. An example of a logarithmic plot of 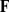 versus 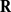 generated from a single iteration of our simulation is shown in Figure 2. Using the textbook definition of 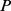-value, the linear slope obtained from the logarithmic plot of 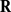 versus 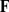 should be approximately one for methods with accurate statistics. To quantify how well 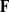 agrees with 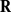 we use four measures: (1) the average weighted sum of squares error (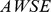), (2) the distance (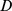) between 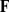 and 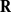, (3) the expected rank E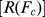, and (4)the expected error of 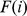. Figure 2 also illustrates what is being computed by the above four measures.

#### Average Weighted Sum of Squares Error

We define the average weighted sum of squares error as
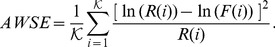
(15)


The weight factor (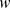), 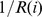, in the above equation was chosen so that each point in the transformed variable domain carries the same contribution to the 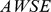. By construction, the 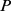-values in the random vector 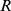 are uniformly distributed between 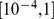. However, once we make the logarithmic transformation, 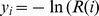, we find the new variable 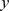 to be exponentially distributed, i.e., 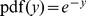. One may thus introduce 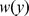, a weight factor making 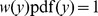, to compensate the non-uniformity in 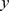. This leads to 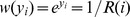, the weight factor used in eq. (15).

#### Angular Distance Between F and R

To compute the distance between 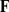 and 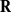, we began by first computing the slope (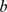) of the logarithmic plot of 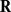 versus 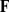 using a weighted least-square regression, which aims to minimize the weighted sum of squares error (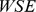)
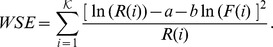



Taking the derivative of the above expression with respect to 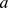 and 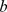 and setting them equal to zero gives the following equations:
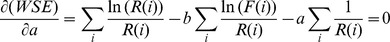
and
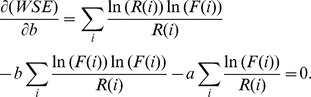



Solving the above two equations simultaneously for a and b gives
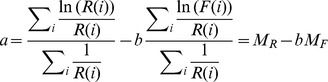
where 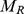 and 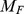 are the weighted average of 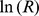 and 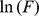 respectively and
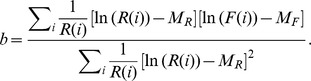



From 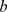 and 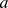, a normalized vector 
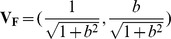
 was computed using the points 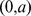 and 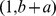 along the regression line. Similarly another normalized vector 
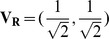
 was obtained using the points 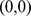 and 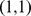 along the ideal line. Finally, the (angular)distance between the two unit vectors 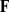 and 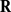 was computed
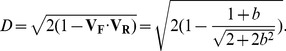
(16)


Methods with accurate statistics are expected to have 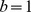 and 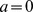. Evidently, 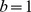 leads to 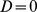 (see eq. (16)). The independence of the angular distance 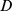 on the intercept parameter 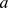 implies that 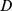 only measures the relative accuracy of the 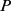-value, not the absolute accuracy. For example, if 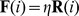, even when the positive constant 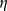 is different from 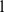, 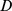 is still zero.

#### Expected Rank E[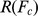]

For iteration 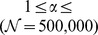, we denote by 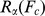 the largest normalized rank whose corresponding reported 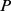-value is less than or equal to a selected cutoff 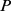-value 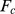. The expected rank E[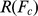] is computed by averaging 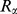 over all realizations and can be written as
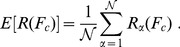
(17)


In the ideal case of absolute accuracy, 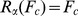. In reality, this is hardly the case and that is why we use the expectation value of 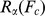 versus 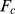 as the measure. For methods with accurate statistics a plot of E[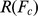] versus 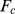 should trace closely the line 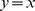.

#### Expected Error of 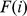


The expected error of 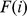 relative to 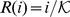 (for 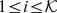) is defined as
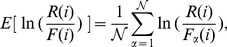
(18)and the standard deviation
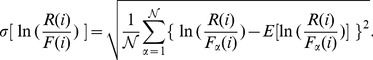
(19)


For methods with accurate statistics, plotting 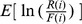 versus 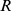 should track the line 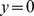 well and have small standard deviations for various 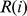.

## Results and Discussion

**Figure 3:**
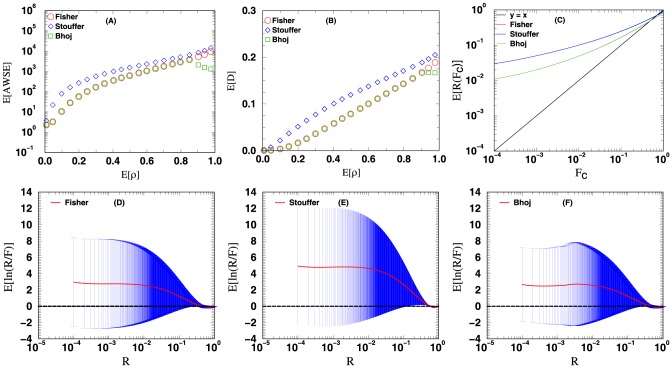
Methods that combine independent 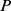-values: Fisher, Stouffer and Bhoj. The curves plotted above are the curves for the four different measures used to evaluate the accuracy of the computed 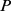-value from combining the 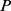-values of 12 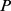
- value vectors.In panel C, note that the Fisher curve (red) is almost completely covered by the Bhoj curve (green). See text for more details.

**Figure 4:**
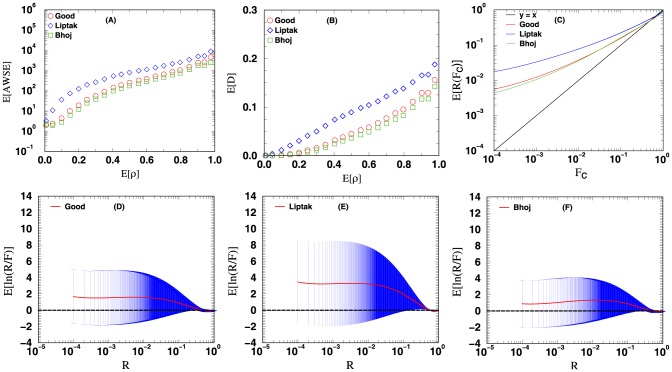
Methods that combine weighted independent 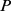-values: Good, Lipták and Bhoj. The curves plotted above are the curves for the four different measures used to evaluate the accuracy of the computed 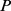-value from combining the 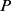-values of 12 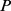-value vectors. See text for more details.

**Figure 5:**
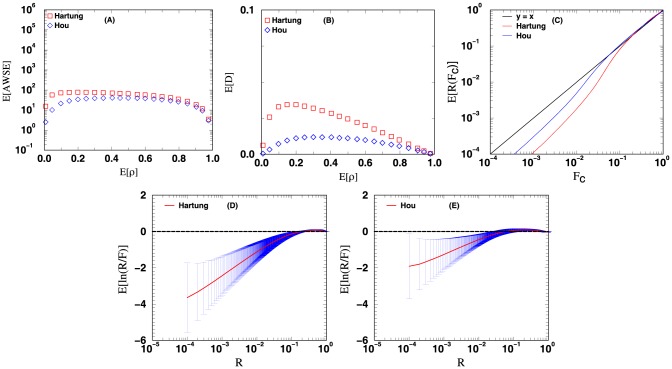
Methods that combine correlated 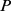-values: Hartung and Hou. The curves plotted above are the curves for the four different measures used to evaluate the accuracy of the computed 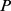-value from combining the 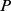-values of 12 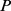-value vectors. See text for more details.

**Figure 6:**
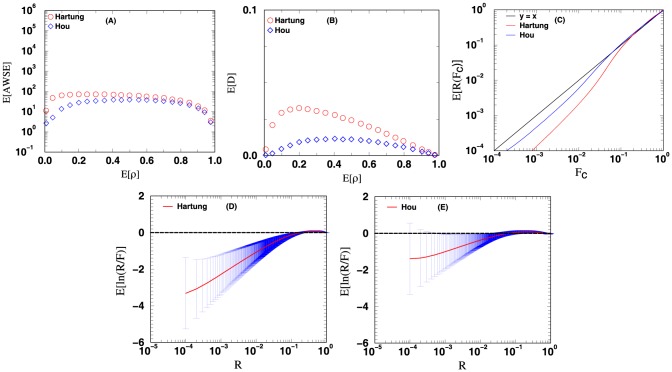
Methods that combine weighted correlated 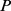-values: Hartung and Hou. The curves plotted above are the curves for the four different measures used to evaluate the accuracy of the computed 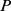-value from combining the 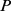-values of 12 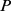-value vectors. See text for more details.

The four measures mentioned in the methods section are used to evaluate the accuracy of the unified 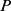-value computed. In Figures 3, 4, 5 and 6, we show the results of combining a list of 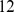 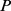-values. The layout of each of these figure is identical. For each method considered, our simulation includes a total of 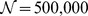 iterations. At each iteration, we generated 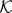 lists, within which the 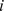
th list is obtained by taking the 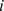 entry of each of the 12 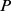-value vectors, 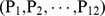. By computing the pairwise correlation (see eq. (8)) among the 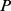-value vectors, one obtains the average pairwise correlation E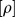 given by eq. (7). Each iteration, generating a 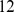-tuples of 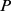-value vectors, thus yields an average correlation 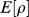.

For Figures 3, 4, 5, 6, the data points in panels A and B respectively display the expected average sums of square errors (E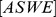) and expected distances (E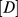) versus E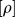. More specifically, every data point plotted with 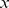-axis value 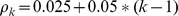 represents an average of 25,000 iterations, each of which has its 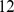-tuple's average correlation 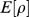 fall in the range of 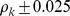. For panels C, D, E and F, each data point plotted is computed using all the 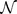 iterations from our simulation. The curves in panel C show the expected number of events with unified 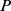-value computed less than or equal to a cutoff value 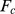. For methods with accurate statistics, by the definition of 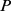-value, a plot of E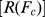 versus 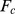 should follow the line 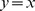. Panels D and E (and F for Figures 3 and 4) display the expected 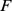 value together with its standard deviation as a function of 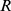. Similar plots for the combination of 4 and 8 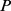-value vectors can be found in File S1.

Figure 3 displays the results for methods that assume the the 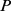-values to be combined are independent: Fisher's (eq. 3), Stouffer's (eq. 1) and Bhoj's (eq. 6) methods. These methods are expected to compute accurate combined 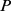-values for E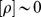, corresponding to the first few data points of panels A and B. The data points in panels A and B show that as E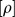 increases so does the E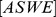 and E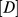, indicating the methods' inadequacy for handling correlation among 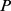-values. All three curves in panel C lie above the 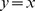 line, indicating that all three methods exaggerate significance when combining correlated 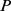-values. The curves in panels D, E and F show that the average value (red solid curve) of 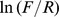 can deviate significantly from 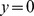 axis with wild fluctuations (error bars shown in blue). Also, a comparison with the plots obtained from combining 4, 8, and 12 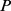-value vectors indicates that the accuracy of the unified 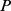-value decreases as the number of 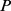-values combined increases from 4 to 12.

Figure 4 shows the results for methods that combine weighted independent 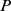-values: Good's (eq. 5), Lipták's (eq. 1) and Bhoj's (eq. 6) methods. These three methods may be viewed as extensions of the previous three methods with 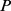-value weighting enabled. Comparison of the panels of Figure 4 with that of Figure 3 shows noticeable improvement on the accuracy of the combined 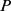-values. Although the accuracy has improved by weighting the 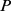-values, the computed 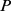-value still differs significantly from the expected value. The observed improvement suggests that weighting 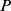-values might weaken the effect of correlation by promoting one 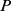-value over the rest in the list of 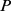-values to be combined. Other studies have also recommended [32], [33] weighting 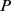-values to improve statistical power. Even though weighting 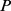-values is recommended, there exists no consensus on how to determine the optimal weights [6], [24]-[26]. This is why in our simulation we have assigned random weights to the 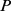-values to be combined. In principle, the accuracy of the computed 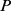-value from the three methods above could be improved by using a different procedure to compute the weights. Such an investigation, although worth pursuing in its own right, is beyond the scope of the current study.

Figure 5 shows the results from using methods designed to combine correlated 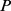-values: Hartung's (eq. 9) and Hou's (eq. 14) methods. The curves in Figure 5 when compared with the curves of Figure 3 and 4 show a significant improvement in the accuracy of the combined 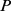-value computed. From the curves of Figure 5 Hou's method seems to be the better performing one, it has a smaller expected error and standard deviation when compared with the curves obtained from Hartung's method. As shown in panel C of Fig. 6, Hou's E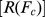 vs 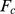 curve also traces reasonable well the line 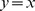, deviating from it only by a factor of about 4.0 for 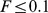.

Finally, in Figure 6 we have the evaluation results of methods that combine weighted correlated 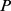-values: Hartung's (eq. 9) and Hou's (eq. 14) methods. When the curves of Figure 6 are compared with that of Figure 5, as before it shows that weighting 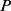-values tends to improve the accuracy of the the computed 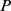-value The curves also show that Hou's method has a larger improvement in accuracy by using weights in comparison to Hartung's method. As articulated earlier and supported by the observed results, there is a possibility that the accuracy of the combined 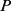-value could be further improved by having a statistically and mathematically rigorous procedure that could render the optimal weights to be used.

In a brief summary, methods designed for combining *independent* 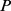-values tend to yield exaggerated 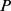-values when used to combining correlated 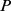-values. On the other hand, most methods designed to handle correlated 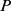-values tend to provide conservative estimates for the unified 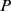-values. The first case can be understood easily since one is effectively using nearly identical evidences to corroborate one another. For the latter case, however, we can not provide an intuitive interpretation except that it might result from the heuristics those methods employed. Weighting 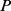-values seems to weaken the effect of correlation. This can be roughly understood as follows. By weighting each of the 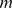 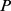-values, only the 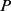-values assigned the highest weights play a role. This increase the likelihood of having the highest weighted 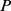-values be nearly independent, thereby reducing the effect of correlations. Not only does it help the methods designed for combining independent 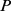-values, it also helps the ones for combining correlated 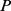-values as most of these methods are heuristic-based and get more accurate results when the correlation is weaker. Based on these results, when the lists of the 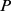-value vectors are complete, it is best to calculate the corresponding pairwise correlations between any two 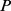-value vectors, introduce weights, and then assign the final unified statistical significance to each hypothesis.

In real applications, however, one is often faced with incomplete lists of 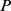-values. That is, one only has the 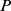-values for the highest ranking hypotheses, not for all hypotheses tested. This prevents one from computing the correlations needed for the formalism for combining correlated 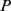-values. In this case, *i.e.*, when combining 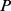-values of unknown correlation, one should exercise caution. Absent the correlation information, a better option might be to use the smallest of the 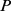-values to be combined and then apply the Bonferroni correction by multiplying the smallest 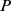-value by 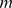, the number of 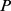-values to be combined. This will guarantee a conserved statistics. However, under this approach, one might run into cases where the smallest 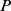-values considered is larger than 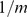, thereby obtaining a corrected 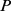-value that is larger than 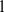. Even if each of the 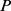-value lists is complete, there are still scenarios not covered in this paper. For example, it is possible that higher order correlations (such as the three-body or four-body) exist among the 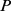-value vectors. We did not consider these cases since we are not aware of any readily available methods designed to deal with such type of higher order correlations.

In conclusion our study recommends that the unified 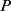-value obtained from combining 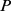-values of unknown correlation should be used with caution to prevent from drawing false conclusions. Results from our study agree with previous investigations [6], [8], [10], supporting the hypothesis that weighting 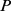-values has the potential to improve the accuracy of the combined 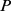-value. However, the important issues of choosing the weights to optimize a method's power and estimating the correlation matrix elements among 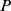-values from small sample sizes remain challenging [34], [35]. Our results also show that when combining independent or weighted independent 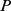-values, Bhoj's method produces more accurate 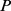-values than other methods tested. In the case when the correlation information is available, among the methods investigated, Hou's method, able to accommodate 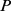-value weighting, seems to be the best performing method.

## Supporting Information

File S1This pdf file contains eight figures showing 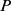-value accuracy evaluation of methods considered in this manuscript when combining 4 and 8 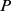-value vectors.(PDF)
