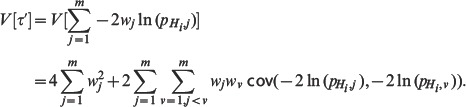# Correction: Accuracy Evaluation of the Unified *P*-Value from Combining Correlated *P*-Values

**DOI:** 10.1371/journal.pone.0103662

**Published:** 2014-07-25

**Authors:** The PLOS ONE Staff

There are multiple errors in Equations 4 and 11. Please see the corrected Equation 4 here.
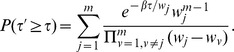



Please see the corrected Equation 11 here.