# Supplementary material for: Accuracy Evaluation of the Unified P-Value from Combining Correlated P-Values
Source: PLoS One. 2014 Mar 24;9(3):e91225. doi: 10.1371/journal.pone.0091225 (PMC3963868; doi:10.1371/journal.pone.0091225)
Supplement: File S1 — This pdf file contains eight figures showing -value accuracy evaluation of methods considered in this manuscript when combining 4 and 8 -value vectors. (PDF) [file pone.0091225.s001.pdf]

Supporting Information: File S1

Gelio Alves and Yi-Kuo Yu*

National Center for Biotechnology Information, National Library of Medicine, National Institutes of Health, Bethesda, Maryland, United States of America

∗ E-mail: yyu@ncbi.nlm.nih.gov

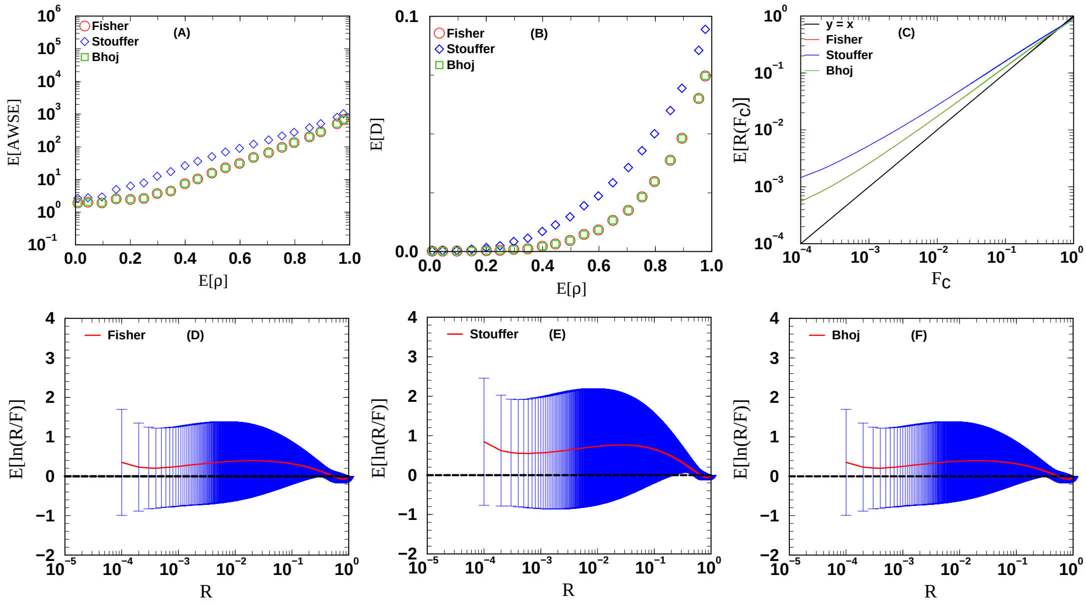

Figure S1. Methods that combine independent P-values: Fisher, Stouffer and Bhoj. The curves plotted above are the curves for the four different measures used to evaluate the accuracy of the computed P-value from combining the P-values of 4 shuffled P- value vectors. In panel C, note that the Fisher curve (red) is almost completely covered by the Bhoj curve (green). See text for more details.

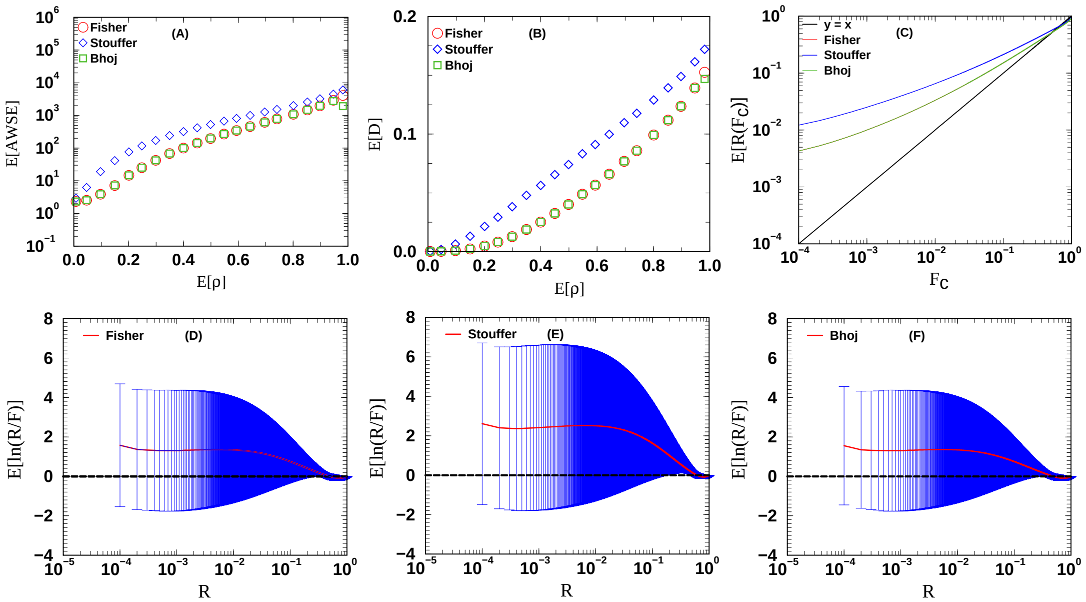

Figure S2. Methods that combine independent P-values: Fisher, Stouffer and Bhoj. The curves plotted above are the curves for the four different measures used to evaluate the accuracy of the computed P-value from combining the P-values of 8 shuffled P- value vectors. In panel C, note that the Fisher curve (red) is almost completely covered by the Bhoj curve (green). See text for more details.

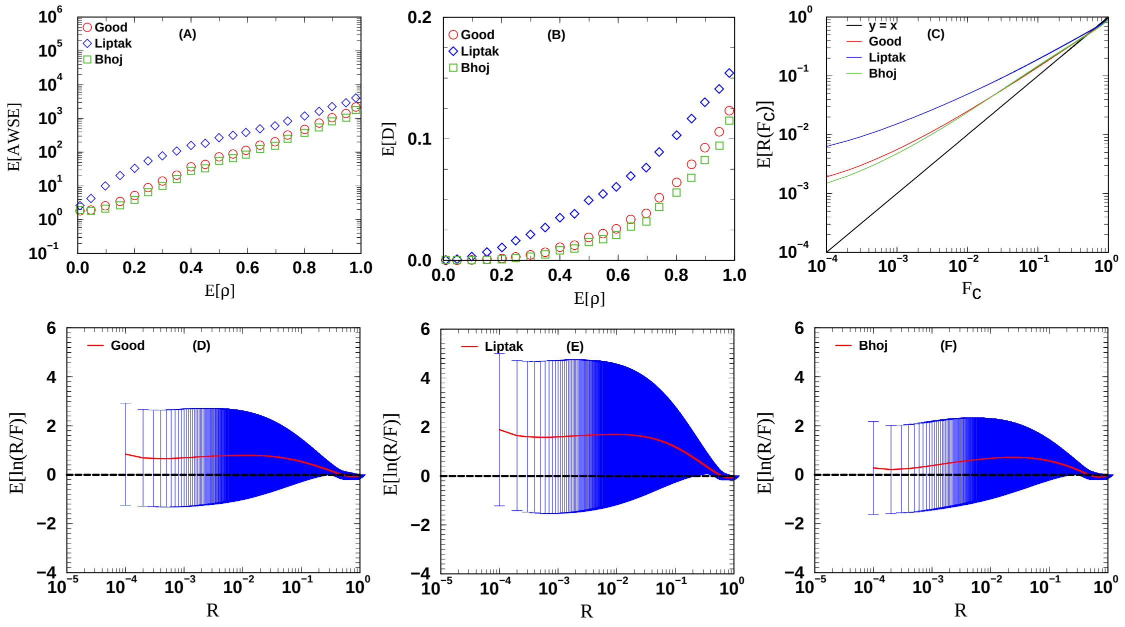

Figure S3. Methods that combine weighted independent P-values: Good, Lipták and Bhoj. The curves plotted above are the curves for the four different measures used to evaluate the accuracy of the computed P-value from combining the P-values of 4 shuffled P-value vectors. See text for more details.

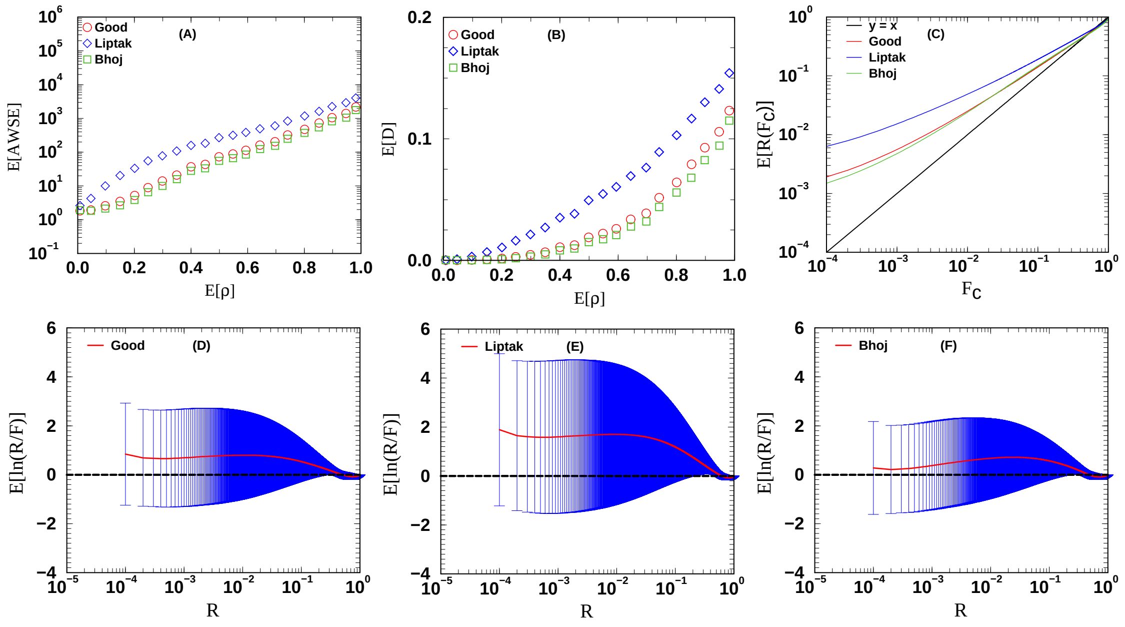

Figure S4. Methods that combine weighted independent P-values: Good, Lipták and Bhoj. The curves plotted above are the curves for the four different measures used to evaluate the accuracy of the computed P-value from combining the P-values of 8 shuffled P-value vectors. See text for more details.

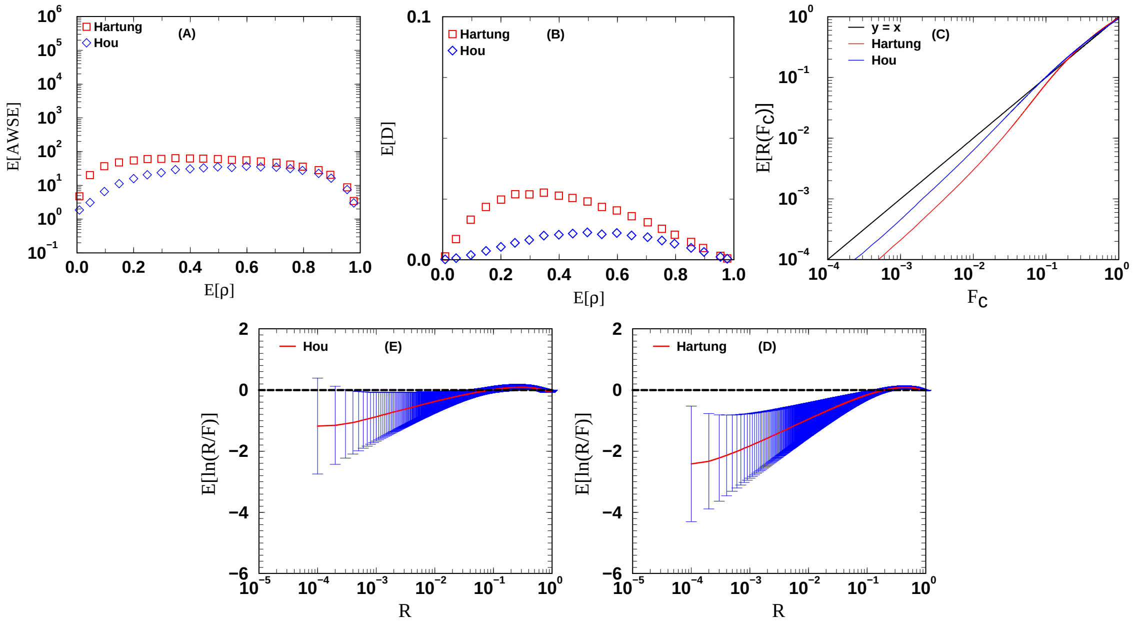

Figure S5. Methods that combine correlated P-values: Hartung and Hou. The curves plotted above are the curves for the four different measures used to evaluate the accuracy of the computed P-value from combining the P-values of 4 shuffled P-value vectors. See text for more details.

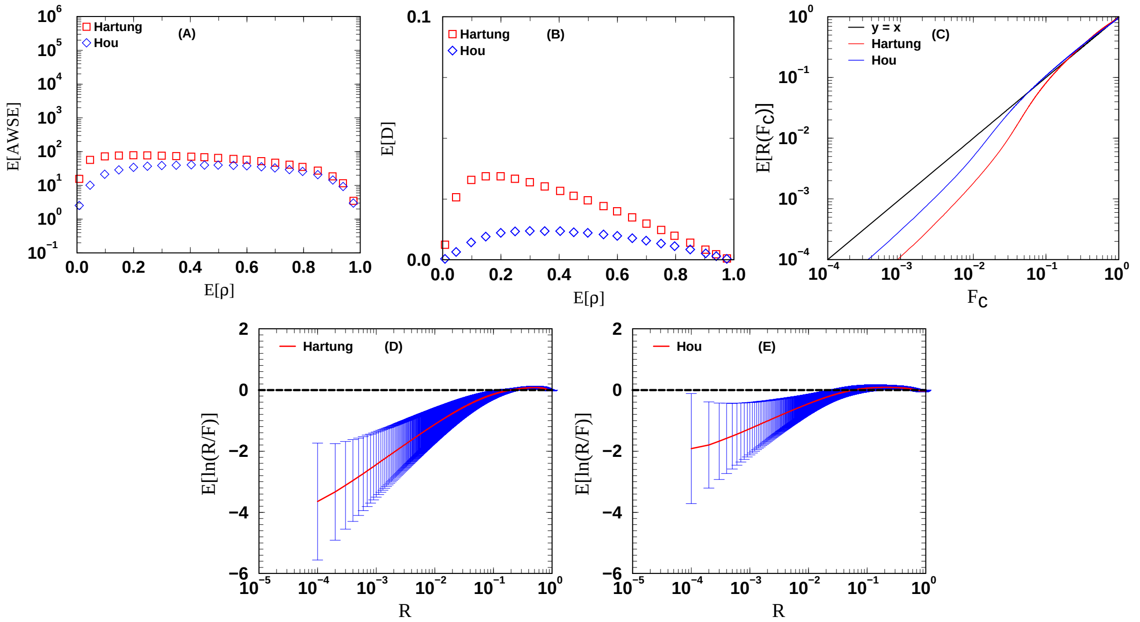

Figure S6. Methods that combine correlated P-values: Hartung and Hou. The curves plotted above are the curves for the four different measures used to evaluate the accuracy of the computed P-value from combining the P-values of 8 shuffled P-value vectors. See text for more details.

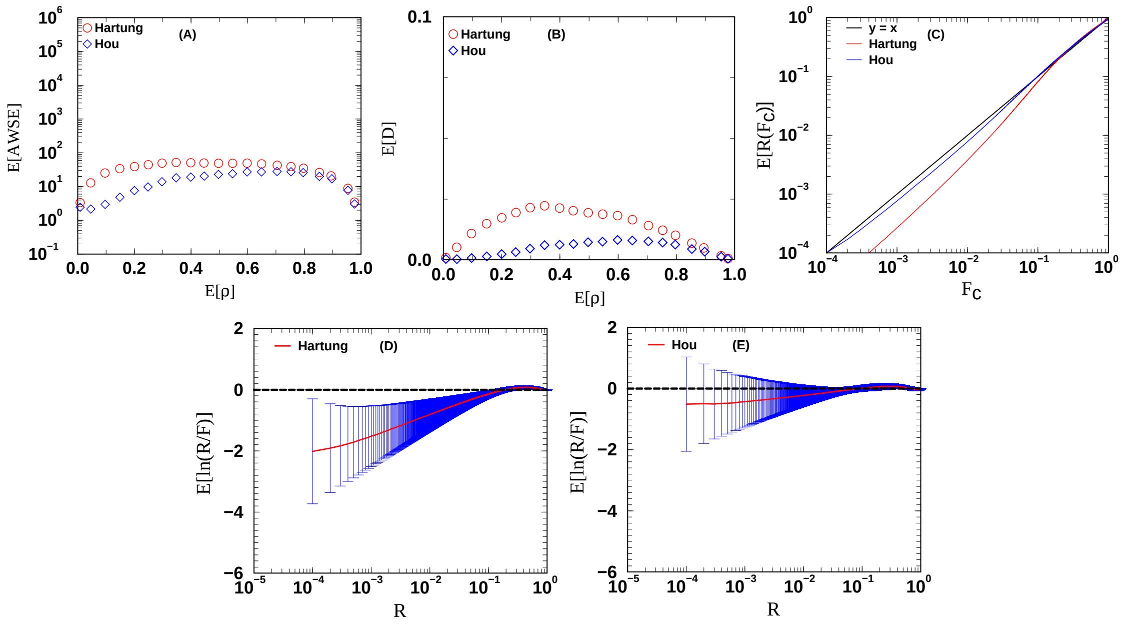

Figure S7. Methods that combine weighted correlated P-values: Hartung and Hou. The curves plotted above are the curves for the four different measures used to evaluate the accuracy of the computed P-value from combining the P-values of 4 shuffled P-value vectors. See text for more details.

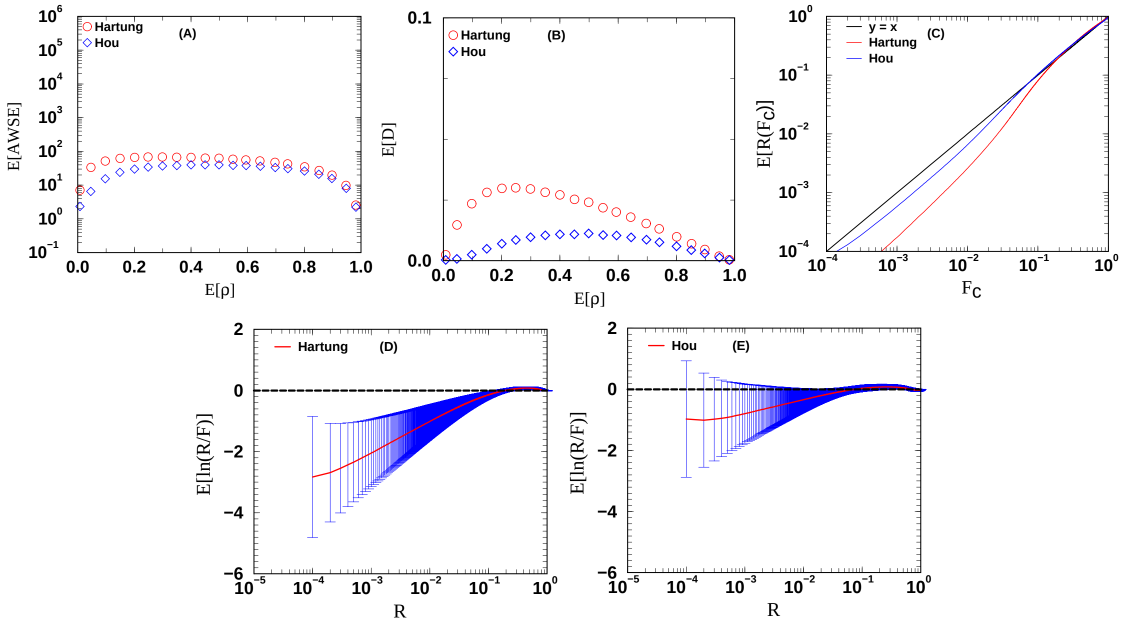

Figure S8. Methods that combine weighted correlated P-values: Hartung and Hou. The curves plotted above are the curves for the four different measures used to evaluate the accuracy of the computed P-value from combining the P-values of 8 shuffled P-value vectors. See text for more details.
